# Analysis of initial sandplay characteristics among university students with different levels of loneliness

**DOI:** 10.1186/s12888-023-05443-y

**Published:** 2023-12-11

**Authors:** Zheng Qiu-Qiang, Li Bo-Lin, Yang Wei-Wei, Zhu Yu, Zhang Qi-Zhe

**Affiliations:** 1 School of Education Science, Huizhou University, Huizhou, China; 2 Institute of Analytical Psychology, City University of Macau, Macao, Macao SAR China; 3 School of Education, Beijing Normal University Zhuhai, Zhuhai, China; 4 Mental Health Education and Counseling Center, Beijing Normal University Zhuhai, Zhuhai, China; 5 Mental Health Education and Counseling Center, Huizhou University, Huizhou, China

**Keywords:** Initial sandplay, Loneliness, University students emotion, Psychological activity, Psychological characteristics

## Abstract

**Background and objective:**

Loneliness is detrimental to mental health, with university students at higher risk of feeling lonely than other population groups. The mental health of college students is a hot topic at present. Despite numerous studies exploring interventions for loneliness among university students. However, little research has explored early psychological manifestations of university students with different levels of loneliness. Despite numerous studies exploring interventions for loneliness among university students, little research has explored early psychological manifestations of university students with different levels of loneliness. Initial sandplay is a good tool to reveal psychological activity. Therefore, our study aims to explore the characteristics of initial sandplay application among university students with different levels of loneliness.

**Methods:**

We recruited 60 volunteers from a university to perform a sandplay experiment from January to April 2021. The UCLA Loneliness Scale measured the levels of loneliness. These 60 participants were divided into the experimental group (*n* = 30) and control group (*n* = 30) according to their levels of loneliness. The experimental group included participants with a scale score of more than 44. Other participants with a scale score of less than 44 belong to the control group. We recorded their sandplay artwork and statistically analyzed it by the Sandplay Process Record Form. Group comparisons were performed using the t-test or Wilcoxon rank-sum test for continuous variables, and the chi-square test or Fisher’s exact test for categorical variables. The logistic regression analysis by forward stepwise method was conducted to analyze the sandplay theme features for loneliness.

**Results:**

Regarding the sandplay tools, the experimental group used fewer transportation tools (*t*=-3.608, *p* < 0.01) and more natural elements (*t* = 2.176, *p* < 0.05) than the control group. Moreover, the experimental group created more natural scenes (χ^2^ = 4.310, *p* < 0.05) and used less of the lower left (χ^2^ = 4.593, *p* < 0.05) and lower right (χ^2^ = 5.934, *p* < 0.05) spaces. With regards to sand changes, the experimental group was less likely than the control group to make substantial changes (χ^2^ = 5.711, *p* < 0.05) and more likely to make almost no changes (χ^2^ = 4.022, *p* < 0.05). In terms of the themes, the experimental group was more likely to exhibit sandplay artwork themes of emptiness (χ^2^ = 8.864, *p* < 0.05) and neglect (χ^2^ = 6.667, *p* < 0.05), and less likely to show themes of energy (χ^2^ = 5.079, *p* < 0.05). In the logistic regression analysis of the sandplay themes, emptiness (OR = 5.714, 95%CI: 1.724–18.944, *p* = 0.003) and neglect (OR = 7.000, 95%CI: 1.381–35.479, *p* = 0.010) were demonstrated a nominal association with high levels of loneliness among both groups (F = 16.091, *p* < 0.01, ΔR^2^ = 0.193), but failed to pass the Bonferroni testing correction (*p* threshold < 0.0025).

**Conclusion:**

University students with higher degree of loneliness do not like to drastic changes and prefer to use natural elements in element selection, while the control group likes to drastic changes and prefers to use transportation tools in element selection. Regression analysis of sandplay theme features revealed emptines and neglect may as significant associated factors for loneliness. We propose sandplay characteristics can help identify university students with different levels of loneliness during psychological evaluations. Therefore, it is important that the school and healthcare systems assist college students in identifying the loneliness through initial sandplay and carrying on the necessary psychological counseling to the lonely student population.

## Introduction

Loneliness is a subjective, negative experience characterized by fewer social contacts than desired or an absence of the expected level of intimacy in relationships [1]. Worldwide, young children and teenagers are commonly affected by loneliness. From 2012 to 2018, school loneliness increased in 36 of 37 countries. In comparison to 2012, over twice as many teenagers worldwide experienced increased levels of school loneliness in 2018. In particular, in 2014–2015, 32.4% of German students felt moderately lonely, and 3.2% felt severely lonely [2]. In 2018, young adults between the ages of 18 and 22 have been identified as the most alone generation in America [3]. Data derived from the Global School-based Student Health Survey of children showed that the overall prevalence of loneliness was 11.7% (95% confidence interval (CI): 10.6–12.7), with significant variations across countries. Girls have a higher prevalence ratio (PR = 1.4, 95% CI: 1.3–1.4) than boys [4]. In Latin America and the Caribbean, it was reported that 18.1% of students are lonely most or all of the time and have no close friends, including 19.9% of girls and 16.2% of boys [5]. Students’ loneliness is associated with more significant anxiety, stress and depression [6–9]. It also has been associated with maladaptive coping styles, sleep problems, lower life satisfaction and suicidal ideation [10–12]. In addition, loneliness has also been related to impaired cognitive health and mental health problems [8, 13–15]. Loneliness impacts brain functionality. Chronic loneliness likely changes the nature and likelihood of social interactions [16–18]. In recent years, due to COVID-19 impact, the prevalence of loneliness among college students has further increased significantly [19–22]. Indeed, lonely individuals also have more unique personalities. Researchers found that lonely individuals were more self-centred, less communicative, and rarely engaged with others. They preferred to talk about subjects unrelated to the interests of their discussion partner.

Mental health educators and practitioners use diverse techniques, such as sandplay, to understand people’s psychological state. Psychologist Dora Kalff developed Sandplay, which is a psychological counselling and therapy technique based on Jungian psychoanalysis. Initial sandplay was the first sandplay therapy. Sandplay therapy began in the 1950s and served as a clinical psychological evaluation and diagnosis tool for adults and children with mental health issues. Sandplay can effectively reflect the specifics of individual mental activity because sandplay is very personalized, and no two sessions or outcomes (sandtrays) are ever the same. After decades of development, sandplay has been applied to the in-depth interpretation of psychological diseases.

In recent years, it has been proved that sandplay plays an important role in the evaluation of mental activity and the intervention treatment of psychological disorders. One study reported significantly different sandplay characteristics between high-family function students and those with low-family function. Samples with low family function utilized fewer animal figurines in their sandplay (*t* = 2.176, *p* < 0.05), had more family scenes (χ^2^ = 4.356, *p* < 0.05), fewer rural scenes (χ^2^ = 4.344, *p* < 0.05), and fewer healing elements (*t* = ™2.336, *p* < 0.05). Specifically, there were fewer instances of connected healing themes (χ^2^ = 7.500, *p* < 0.05), in-depth healing themes (χ^2^ = 5.455, *p* < 0.05), and more hindered trauma elements (χ^2^ = 4.812, *p* < 0.05). The sandplay characteristics were utilized as predictors of group membership in a logistic regression analysis. Themes of impeded trauma (B = ™2.030) and connected healing (B = 1.765) were found to be significant predictors of group membership by forward stepwise analysis (F = 17.784, *p* < 0.01, ΔR^2^ = 0.214) [23]. Sandplay characteristics can help identify low family functioning in adolescents during psychological evaluations. One exciting study suggest that children’s sandplay confirms the traumatic nature of immigration detention and reveals their conflicting understanding of detention and their migration [24]. The results are contextualized by a description of detention conditions and the psychiatric symptoms associated with immigration incarceration [24]. The study emphasizes the need for additional research on the impact of immigration detention on children’s mental health, as well as the importance of refugee children’s voices in guiding policy change. Additionally, it has been demonstrated that sandplay has a significant therapeutic influence on a variety of mental disorders. Sandplay therapy can reduce anxiety, withdrawal, and social and behavioral issues in school-aged children with chronic conditions, as well as alleviate symptoms of anxiety and melancholy in their caregivers [25]. One study suggested that sandplay therapy improves ADHD symptoms, such as hyperactivity, impulsivity, and inattention, through symbolic expression, play, and mindfulness [26]. The research also revealed that image-sandplay therapy can alleviate the symptoms of autism in children and is conducive to promoting mental health and increasing SWB. Sandplay therapy is a feasible and effective rehabilitation method [27]. Furthermore, studies have demonstrated that sandplay therapy can be used as a psychological intervention therapy for a number of clinical illnesses, such as systemic lupus erythematosus and an autism spectrum disorder [28–30].

However, there were limited studies of sandplay’s application in the description of psychological activity of loneliness in university students. University students with different levels of loneliness may have various mental activities feature. Sandplay can be used to map these mental activities in greater detail and even reveal subtle psychological aspects that were easily overlooked. Based on the interpretation of inner mental activities by sandplay, appropriate psychological intervention can be conducted to improve students’ loneliness subsequently. Initial sandplay as the first time sandplay, operated by the same rules while lack of subsequent sandplay operation. Therefore, our research aims to explore the characteristics of different levels of loneliness of university students in initial sandplay, and to establish the relationship between the characteristics of sandplay and loneliness of university students. The completion of the research is helpful to further use external intervention tool to interpret the psychological issues of university students. The findings of this study will provide a reference for diagnosing and evaluating loneliness among university students.

## Research subjects and methods

This is a cross-sectional study. We recruited 74 university students to complete a sandplay experiment between January to April 2021, according to a random sample based on college. Participants first completed the UCLA loneliness scale online. Participants ranged from age 18 to 21 (MD = 19.55; SD = 0.81). There were 38 females and 36 males. The inclusion criteria was the students in Beijing Normal University, Zhuhai college, aged between 18 and 23 years. The exclusion criteria were: mental illnesses (depression, mania, moderate or severe anxiety), currently receiving psychological intervention or has previously received psychological intervention, advanced cancers. Four participants were reported previously received psychological intervention, and three participants reported different degrees of psychological disorders such as anxiety and depression. One participant was being treated for malignant lymphoma. Six participants temporarily dropped out of the trial before the trial, therefore, 14 volunteers did not participate in the experiment finally. We included 60 qualified participants, and completed 60 valid questionnaires. The included subjects ranged from age 18 to 21 (MD = 19.55; SD = 0.81), mean age is 19.52, and the SD is 1.03, 28 subjects were male, and 32 subjects were female. There were 19 freshmen, 23 sophomores, 12 juniors and 6 seniors. We divided sixty participants into an experimental group (EG) and a control group (CG) according to a total scale score. We ranked the loneliness scale scores of the study participants, assigning cutoff value according to the median of the study participants’ loneliness scale scores. We recognized participants with a total scale score of more than 44 points (based on the median of the study participants’ UCLA Loneliness Scale) as having high levels of loneliness. The experimental group contained high-loneliness students with scores of more than 44. The control group included low-loneliness students with scores of less than 44. There were thirty participants, respectively, in the experimental and control groups. 14 volunteers absent from the final experiment accepted other mental health activities organized by the university.

The Ethics Committee of Beijing Normal University Zhuhai has approved the study. The ethical approval number is 2020-89. We received written informed consent from participants in this study. Legally Authorized Representatives of illiterate participants provided informed consent for the study. The study was conducted following the Declaration of Helsinki.

## Research tools

### UCLA loneliness scale

The UCLA Loneliness Scale, developed by Russell (1988), is a self-report measure utilized extensively in China for loneliness research. The scale consists of 20 items that assess subjective experiences of loneliness and social isolation. Participants rate each item using a 4-point Likert scale ranging from 1 (Never) to 4 (Often) for a total score of 80. The scale has high reliability and validity for college students, with an internal consistency coefficient α = 0.94.

### Sandplay consulting room

A standard sandplay consultation room was established and consisted of a classic sandbox (size: 57 cm high*72 cm wide*7 cm deep) [6]. The blue-painted interior of the sandbox contained four sand racks and clean fine sand and displayed more than 800 different types of sandplay tools. Recording tools included cameras, recording pens, and sandplay record tables.

### Sandplay scoring items and evaluation indicators

#### Use of sandplay tools and coding

We classified the sandbox tools according to Cai Baohong’s classification. They included several elements, such as figures, animals, plants, buildings, natural ingredients, weapons, daily necessities, religion, transportation, and others. We recorded the frequency of each type.

#### Scene types

Scene types included family, rural, transportation, battle, nature, zoo, and unreal. When a scenario occurred, it was recorded as “1,” and when it did not, it was recorded as “0”.

#### Use of sand

The sand change was divided into almost no moving sand, moderate moving sand, and large moving sand, and recorded as “0,” “1,” and “2,” respectively.

#### Sandplay theme features

According to Cai Chenghou’s classification, sandplay themes are divided into 10 trauma themes (chaos, emptiness, split, limitation, neglect, injury, threat, burial, inversion, obstruction) and 10 healing themes (conformity, connection, flow, depth, nurturing, rebirth, energy, spirituality, centralization, change). If the theme appeared, it was coded as “1,” and if it did not appear, it was coded as “0”.

#### Use of sand table works in the field of space

The sand table was divided into nine grids—upper left, left center, lower left, middle-upper, middle lower, middle, upper right, right center, and lower right. To record whether the area was used, the location was coded “1,” if used, and “0,” if it was not used.

#### Experimental procedure

A one-on-one investigation was conducted in the university’s sandbox consultation room. The main tester introduced the sandplay game and explained the rules to participants. Every participant accepted identical instructions to complete the initial sandplay artwork within 50 min. The main tester remained silent throughout this proceeding and recorded the participant until completion. After completion, the primary tester and the participant discussed the sandplay. This conversation lasted for an average of about 20 min. After each participant left, the main tester took photos of the artwork, cleaned the sandplay equipment, and recorded the characteristics of each participant’s sandplay works based on the pictures.

### Data processing and statistical analysis

The researcher organized the results of the experimental groups and control groups. They analyzed the data using version 25 of the statistical software SPSS. For sandplay tools usage, Continuous variables that exhibited a normal distribution were documented as the mean ± standard deviation (SD). Otherwise, they were documented as medians with upper and lower quartiles. Categorical variables were documented as frequencies with percentages. Group comparisons were performed using the t-test or Wilcoxon rank-sum test for continuous variables, and the chi-square test or Fisher’s exact test for categorical variables. The logistic regression analysis by forward stepwise method was conducted to analyze the sandplay theme features for loneliness. The two-tailed *p*-value of less than 0.05 indicated a statistical significance, and in the logistic regression analysis, Bonferroni correction *p*-value of less than 0.0025 indicated a statistical significance.

## Results

### Usage of sandplay tools and sandplay scene type situation setting

In the initial sandplay of the EG, the number of figures, animals, plants, buildings, connecting objects, daily necessities, weapons, and sandplay tools was less than that in the CG. However, there was no significant difference between the two groups. The EG appeared more often than the CG in transportation tools (*t* = -3.608, *p* < 0.01) and natural elements (*t* = 2.176, *p* < 0.05). This difference was statistically significant and statistically meaningful, as shown in Table 1. The EG presented significantly more natural scenes compared to the CG. The EG was also considerably more willing to create natural scenes. The difference was statistically significant (*p* < 0.05). Although the EG showed fewer traffic scenes, the difference was not statistically significant (*p* > 0.05), as shown in Table 2.

**Table 1:** Type of sandplay tools and frequency during initial sandplay

Type	Experimental group	Control group	*t*	*P*
Figures	4.733 ± 2.420	5.600 ± 1.831	1.564	0.123
Animals	2.933 ± 1.760	3.633 ± 1.691	1.571	0.122
Plants	5.767 ± 2.622	6.367 ± 3.168	0.799	0.427
Buildings	2.867 ± 2.300	2.567 ± 1.135	-0.641	0.524
Transportation	1.700 ± 1.291	2.933 ± 1.311	-3.672	0.000
Religion	0.233 ± 0.626	0.533 ± 0.776	1.648	0.105
Natural element	2.467 ± 1.570	1.567 ± 1.633	-2.176	0.034
Daily necessities	2.167 ± 2.069	2.467 ± 2.460	0.511	0.611
Weapon	0.267 ± 0.980	0.200 ± 0.805	-0.288	0.774
Other	5.167 ± 3.018	4.800 ± 2.524	0.510	0.612
Total	28.300 ± 5.926	30.667 ± 6.315	-1.497	0.140

Continuous variables are reported as mean ± SD.

**Table 2:** Scene type analysis status

Scene Type	Experimental group (*n* = 30)	Control group (*n* = 30)	χ [31]	*P*
Family	5 (16.667)	8 (26.667)	0.844	0.347
Transportation	10 (33.333)	17 (56.667)	3.300	0.069
Natural	18 (60.000)	9 (30.000)	5.454	0.020
Battle	0 (0.000)	1 (3.333)	1.017	0.313
Unreal	2 (6.667)	0 (0.000)	2.069	0.492
Zoo	1 (3.333)	3 (10.000)	1.071	0.301
Rural	1 (3.333)	1 (3.333)	0.000	1.000

Categorical variables are showed as *n* (%)

### Sand table spatial domain usage and the use of sand

Concerning the two spatial domains in the lower right, the EG demonstrated significantly less utilization than the CG (*p* < 0.05), as shown in Table 3. Compared to the CG, the EG obviously presented more cases of almost unchanged sand and fewer cases of changed sand (*p* < 0.05), in addition, in the term of drastic changes, CG obviously prone to drastic changes, as shown in Table 4.

**Table 3:** Space domain differences between the two groups

Spatial domain	Experimental group (*n* = 30)	Control group (*n* = 30)	χ [31]	*P*
Upper left	21(70.000)	23(76.667)	0.341	0.559
Left center	20(66.667)	24(80.000)	1.364	0.243
Lower left	15(50.000)	23(76.667)	4.593	0.032
Middle upper	20(66.667)	26(86.667)	3.354	0.067
Middle	29(96.667)	30(100.000)	1.017	0.313
Middle lower	17(56.667)	20(66.667)	0.635	0.426
Upper right	25(83.333)	22(73.333)	0.884	0.347
Right center	22(73.333)	19(63.333)	0.693	0.405
Lower right	15(50.000)	24(80.000)	5.934	0.015

Categorical variables are showed as *n* (%)

**Table 4:** Sand Use Between the Groups

		Experimental group (*n* = 30)	Control group (*n* = 30)	χ [31]	*P*
Changes to the sand	Almost unchanged	12(40.000)	5(16.667)	4.022	0.045
	Slight change	11(36.667)	9(30.000)	0.300	0.584
	Drastic change	7(23.333)	16(53.333)	5.711	0.017

Categorical variables are showed as *n* (%)

### Regression analysis of sandplay theme features with high loneliness

In the comparison of CG, the EG showed more emptiness (χ^2^ = 8.864, *p* < 0.05) and neglect (χ^2^ = 6.667, *p* < 0.05) in terms of traumatic themes and fewer healing themes of energy (χ^2^ = 5.079, *p* < 0.05). These differences were statistically significant, as shown in Table 5. We identified the score of students with high loneliness as the dependent variable (If > 44 points) and determined the sandplay themes as the associated variable. The sandplay theme is a value in the two-point format. The sandplay theme feature’s presence was classified as “1.” The number “0” denotes the lack of a sandplay theme feature. We conducted the logistic regression analysis by forward stepwise method. The regression analysis results showed emptiness (*B* = 1.817, *t* = 2.766, OR = 5.714, 95%CI: 1.724–18.944, *p* = 0.003) and neglect (B = 2.050, *t* = 2.294, OR = 7.000, 95%CI: 1.381–35.479, *p* 0.010) as significant associated factors, as shown in Table 6. We obtained the regression equation Y = -0.960 + 1.817* × *_*1*_ + 2.050* × *_*2*_. Y is the loneliness score. *X*_*1*_ is emptiness, and *X*_*2*_ is neglect. The overall results of the regression model were: F = 16.091, *p* < 0.01, △*R*^*2*^ = 0.193. But this statistically significant failed to pass the Bonferroni testing correction (*p* threshold < 0.0025).

**Table 5:** Topic characteristics analysis of sandplay

Trauma theme	EG (*n* = 30)	CG (*n* = 30)	χ [31]	*p*	Healing theme	EG (*n* = 30)	CG (*n* = 30)	χ [31]	*P*
Chaos	5(16.667)	2(6.667)	1.456	0.228	Connection	5(16.667)	10(33.333)	2.222	0.136
Emptiness	16(53.333)	5(16.667)	8.864	0.003	Flow	8(26.667)	10(33.333)	0.317	0.573
Split	3(10.000)	2(6.667)	0.218	0.640	Energy	19(63.333)	25(83.333)	5.079	0.002
Limitation	5(16.667)	4(13.333)	0.131	0.717	Depth	5(16.667)	8(26.667)	0.844	0.347
Neglect	10(33.333)	2(6.667)	6.667	0.010	Rebirth	5(16.667)	6(20.000)	0.111	0.740
Burial	2(6.667)	3(10.000)	0.218	0.640	Nurturing	3(10.000)	5(16.667)	0.577	0.448
Injury	8(26.667)	3(10.000)	2.783	0.095	Change	4(13.333)	2(6.667)	0.741	0.389
Threat	4(13.333)	4(13.333)	0.000	1.000	Spirituality	3(10.000)	3(10.000)	0.000	1.000
Obstruction	5(16.667)	2(6.667)	1.456	0.228	Centralization	2(6.667)	3(10.000)	0.218	0.640
Inversion	2(6.667)	4(13.333)	0.741	0.389	Conformity	7(23.333)	13(43.333)	2.700	0.100

Categorical variables are showed as *n* (%)

**Table 6:** Regression analysis of sandplay theme features with high loneliness

Variable	Logistic regression analysis	
	OR (95% CI)	*P* value
Chaos	2.800 (0.498-15.7349)	0.424
Emptiness	5.714 (1.724–18.944)	0.003
Split	1.555 (0.241–10.050)	1.000
Limitation	1.300 (0.313–5.404)	0.717
Neglect	7.000 (1.381–35.479)	0.010
Burial	0.643 (0.100-4.153)	1.000
Injury	3.273 (0.774–13.833)	0.095
Threat	1.000 (0.226–4.431)	1.000
Obstruction	2.800 (0.498–15.735)	0.424
Inversion	0.464 (0.078–2.751)	0.671
Connection	0.400 (0.118–1.360)	0.136
Flow	0.727 (0.240–2.206)	0.573
Energy	0.345 (0.103–1.163)	0.454
Depth	0.550 (0.157–1.931)	0.347
Rebirth	0.800 (0.215–2.972)	0.740
Nurturing	0.556 (0.120–2.569)	0.706
Change	2.154 (0.363–12.764)	0.671
Spirituality	1.000 (0.185–5.403)	1.000
Centralization	0.643 (0.100-4.153)	1.000
Conformity	0.398 (0.131–4.153)	0.100

## Discussion

This study was based on university students population that included 60 participants. To the best of our knowledge, this is the first initial sandplay study to evaluate loneliness in university students. The coding of the features of sandplay showed that the university students with higher degree of loneliness do not like to drastic changes and prefer to use natural elements in element selection, while the control group likes to drastic changes and prefers to use transportation tools in element selection. Regression analysis of sandplay theme features revealed emptiness and neglect as significant associated factors for loneliness. The above results indicate that sandplay theme features helps to identify the personal trait of loneliness in university students.

### Use of sandplay tools and scenario type sandplay

Sandplay is a Western psychotherapeutic technique based on the basic theory of Jungian analytical psychology and the philosophical ideas of traditional Chinese culture [32]. The sand frame and sand tools correspond to all things in nature, and the sand box and sand can be understood as a projection of the natural and social environment that the participant is exposed to [33].

The participant in front of the sandplay can fully express his or her thoughts, consciousness and experiences through the projection of the sandplay work. According to the Jungian/Projection theory, the images in sandplay are more concrete and tangible than invisible and intangible [34]. In the hermeneutics of Sandplay process, figure and animal sandplay tools mean interpersonal communication willingness [34]. Natural ingredients such as rocks, stones, shells and gemstones can reflect inner optimism or pessimism, rocks and stones reflect inner pessimism, and shells and gemstones reflect inner optimism [35]. In hermeneutics of Sandplay process, vehicles represent the willingness to build a network of human relationships [34]. In this study, the results showed no significant difference between the EG and the CG in the use of figure and animal sandplay tools. This finding suggests that although university students with high loneliness may have subjective feelings of loneliness, they may desire social interaction and integration. Therefore their behaviour does not necessarily reject these key factors. This is not the same as participants with autism spectrum disorders, who tend to have internal resistance to social interaction and integration, with restricted interests, activity and behaviors [36].

In the initial sandplay, the EG used fewer transportation sandplay tools and presented fewer traffic scenes than the CG. This phenomenon may reflect the absence of communication and poor social interaction. After carefully analyzing the traffic scenes presented by participants in the sand table, compared with the CG, the EG used more obstacles in arranging transportation sandplay tools, such as roadblocks. One viewpoint implies that EG participants are deficient in social connection and communication. This reflects the participants’ lack of intrinsic drive to actively seek interpersonal communication and build interpersonal networks. From a different perspective, this group’s propensity for encountering or proactively erecting obstacles may indicate that they were indeed more prone to feeling lonely. Consistent with the results, EG presented more natural scene types in sandplay situations. Further exploration revealed that the EG used more rock and stone sandplay tools, while the CG used more shells and gemstone sandplay tools. The natural elements used by the EG were more monotonous in color and more extensive in volume, while the natural ingredients used by the CG were more colorful and richer in style. When sharing the sandplay artworks, participants in the EG self-reported that the stone symbolized their anxiety to some extent. At the same time, the CG perceived that shells and gems represented life energy. Although university students with high loneliness were willing to communicate with others, they found it difficult to rid themselves of self-doubt and project vitality. In addition, in the initial sandplay artworks, the results revealed that participants with high levels of loneliness tended to arrange tables and chairs for communication but rarely placed character sand figures near them. Based on this finding, we speculate that university students with high levels of loneliness may want to interact socially but lack the ability. In reality, when people transfer bad interpersonal relationships to a new environment, it inevitably causes a sense of frustration. As a result, experiencing failures can strengthen one’s defense mechanism towards others, leading to mental disengagement that exacerbates feelings of loneliness and makes interpersonal communication uncomfortable.

### The use of sandbox space and sand usage

The spatial configuration in sandboxes has symbolic meaning. Analysis of this spatial symbolism can achieve a better understanding of the work. The upper part of the sandbox represents the conscious and spiritual world, while the lower left corner represents instincts and the creative nature [34]. During the development process from the inner to the external world, this field is frequently used. The lower right corner is more consciously skewed to the earth, containing symbolic meanings such as personal relationships with one’s mother and original attachment or bonding [34]. Conversely, the upper right corner represents socialization and relationships with peers and family members and is associated with individual consciousness [34]. In this study, the EG used significantly less space in the lower left and right regions than the CG. Combining the symbolic meaning of sandplay for analysis, university students with high levels of loneliness may have experienced weak social support and frequent frustration during interpersonal communication. These encounters most likely caused them to become more focused and have distinct life objectives. However, the lack of psychological energy weakens the possibility of developing good interpersonal interactions in the future.

Sandplay provides a free and protected space for its creators. The use of clean and delicate sand offers an opportunity for relaxation and the release of internal tension. The sand modification degree and searching for the blue bottom surface can indicate creativity, self-awareness, and internal motivation. In this study, the EG used significantly less sand than the CG participants, which means weak internal motivation. In the process of sandplay game-making, university students with high levels of loneliness failed to showcase their creativity to build mountains with sand, build rivers, and dig channels. To a certain extent, this reflects their unwillingness to change their behavior, which may also be a cause of their increased sense of loneliness.

### Sandplay theme characteristics

The Healthy Minds Study 2020 included 30,529 individuals from the Fall semester cohort of the 2020 Healthy Minds Study. It reported that loneliness was significantly associated with increased psychotic experiences (odds ratio, 1.32; 95% CI, 1.29–1.36), after adjusting for age, gender identity, race/ethnicity, sexual orientation, and international student status [37].

Logistic regression analysis of sandplay theme characteristics showed that emptiness and neglect are risk factors for university students with high levels of loneliness. Throughout the investigation, the EG participants frequently spent much time deciding which sandplay tools to pick. However, they were hardly able to choose one. This ambivalent state indicated uncertainty and hesitancy. Numerous studies confirm these feelings of loneliness, emptiness, coldness, and lack of vitality [38–40]. To some extent, it suggests that university students with high loneliness lack motivation and experience engaging with the outside world, thus displaying emptiness. Collectively, university students with high loneliness are often perceived as an invisible group, easily ignored, rejected, and misunderstood. Therefore, selecting neglected scene themes indicated a scene of isolation. Being overlooked can make university students with high loneliness more withdrawn and self-protective. The adverse effect intensifies their loneliness. Moreover, the sandplay themes of the EG were unlikely to appear in the healing theme. Energy represents vitality, which provides richness and dynamism in sandplay artworks. The EG’s low energy themes imply that university students who experienced high loneliness lacked mental stamina. It may be related to rigidity in previous interpersonal interactions, self-and other perceptions, lack of experience in interpersonal communication, and absence of social support. Lack of essential contact with the outside world may cause such students to lack fresh psychological energy influxes, hence showing fewer examples of the energy healing themes in sandplay artworks.

### Research implications and applications

Previous studies focused primarily on the subjects’ symptoms, such as the initial sandplay characteristics of high school students with depression and individuals with obsessive-compulsive disorder. They may have been ignored in the research process. From the perspective of loneliness, this study hopes to analyze and explain the initial sandplay from different perspectives.

This study reveals that university students with high levels of loneliness use substantially less transportation sand and far more natural elements in their initial sandplay. Regarding the sandplay theme characteristics, university students with low levels of loneliness presented more traumatic themes of emptiness and neglect and less healing themes of energy. Concerning the use of space, the most common was fewer left and right areas. This study demonstrates that university students who experience significant degrees of loneliness exhibit typical behavior in the areas of sand type, theme, scene type, space use, and sand modification during the first sandplay. This discovery contributes to the diagnosis of the function and utility of clinical psychological assessment for identifying the loneliness of university students.

This study attempts to analyze the initial sandplay to find out the types and quantities of sandplay. The scene presented the theme of the sandplay, the usage of sand, and other dimensions used by university students with low and high degrees of loneliness in their sandplay works. Psychological teachers can conduct corresponding psychological counseling or intervention through this feature to improve their current psychological state and discover their core problems as soon as possible.

The analysis of sandplay works must be combined with a detailed examination of the psychological development characteristics of the participants, who are university students. They are in the physical and mental development stage and have unique physiological and psychological aspects of this stage, which are related to the physical and mental aspects of children and adults. Therefore, consultants need to analyze their sandplay works according to the characteristics of high school students.

#### The strengths and weaknesses of the study

At present, there are still few studies on the assessment of loneliness of college students. As a simple tool, we have confirmed for the first time that the initial sandplay can draw the mental activity psychogram of different loneliness of college students. This discovery promotes the application of the initial sandplay in the interpretation of loneliness of college students. In addition, the current study is the equal number of males and females participating which allows for a more gender-specific view on participants’ health.

Initial sandplay can be useful for evaluating university students’ loneliness levels. This period is marked by stress and anxiety as it represents a watershed period in the lives of most young people. Accordingly, an increased understanding of loneliness factors would be helpful for the evaluation and assessment of these cases. Although numerous studies have explored the issues of loneliness among this demographic, this study also contributes to the field. However, we acknowledge its limitations, such as the research design and cultural issues. These limitations would hinder its applicability to other populations. Nevertheless, given the varying degrees of loneliness this group encounters, we hope our research may help offer alternate approaches for evaluation.

### The limitations of this study

However, the study also faces limitations. First of all, it had the limitation of convenience sampling, which affects the generalizability of the findings. Second, the sample sizes were small and the dropout rate is high (14/74, 18.9%), increasing the risk for selection bias. Our sample consisted of participants studying at Beijing Normal University, Zhuhai college universities, and thus our data may not be generalized to other (student) populations. Third, the current study is based on a cross-sectional investigation, the cross-sectional design of the study does not prove causality relationships and there is a lack of baseline assessment data before the this research. Fourth, the applied research on the initial Sandplay is still relatively limited at present. Therefore, the evaluation of it has not been confirmed by other studies. We hope that there will be more studies in the future to further validate our findings.

## Conclusion

University students with higher degree of loneliness do not like to drastic changes and prefer to use natural elements in element selection, while the control group likes to drastic changes and prefers to use transportation tools in element selection. Regression analysis of sandplay theme features revealed emptines and neglect as significant associated factors for loneliness. We propose sandplay characteristics can help identify university students with different levels of loneliness during psychological evaluations. Therefore, it is important that the school and healthcare systems assist college students in identifying the loneliness through initial sandplay and carrying on the necessary psychological counseling to the lonely student population.

## Data Availability

The original contributions presented in the study are included in the article. Further inquiries can be directed to the corresponding authors.
